# Circularly polarized differential intra-oral antenna design validation and characterization for tongue drive system

**DOI:** 10.1038/s41598-023-36717-w

**Published:** 2023-06-19

**Authors:** Sarita Ahlawat, Binod Kumar Kanaujia, Karumudi Rambabu, Ildiko Peter, Ladislau Matekovits

**Affiliations:** 1 School of Computational and Integrative Sciences, Jawaharlal Nehru University, New Delhi, 110067 India; 2 Dr. Ambedkar National Institute of Technology, Jalandhar, 144011 India; 3 Department of Electrical and Computer Engineering, University of Alberta, Edmonton, AB T6G 2V4 Canada; 4 Department of Industrial Engineering and Management, Faculty of Engineering and Information Technology, George Emil Palade University of Medicine, Pharmacy, Science, and Technology of Targu Mures, 540139 Târgu-Mureş, Romania; 5 Department of Electronics and Telecommunications, Politecnico di Torino, 10129 Turin, Italy; 6 Department of Measurements and Optical Electronics, Politehnica University Timisoara, 300223 Timisoara, Romania; 7 Instituto di Elettronica e di Ingegneria dell’informazione e delle Telecomunicazioni, National Research Council of Italy, 10129 Turin, Italy

**Keywords:** Biomedical engineering, Electrical and electronic engineering

## Abstract

Assistive devices are becoming increasingly popular for physically disabled persons suffering tetraplegia and spinal cord injuries. Intraoral tongue drive system (iTDS) is one of the most feasible and non-invasive assistive technology (AT), which utilises the transferring and inferring of user intentions through different tongue gestures. Wireless transferring is of prime importance and requires a suitable design of the intra-oral antenna. In this paper, a compact circularly polarized differential intra-oral antenna is designed, and its performance is analysed within heterogeneous multilayer mouth and head models. It works at 2.4 GHz in the Industrial, Scientific, and Medical (ISM) band. The footprint of the differential antenna prototype is 0.271 λ_g_ $\times$ 0.271 λ_g_ $\times$ 0.015 λ_g_. It is achieved using two pairs of spiral segments loaded in diagonal form near the edges of the central rotated square slot and a high dielectric constant substrate. Its spiral-slotted geometry further provides the desired swirling and miniaturization at the desired frequency band for both mouth scenarios. Additionally, corner triangular slits on the radiating patch assist in tuning the axial ratio (< 3 dB) in the desired ISM band. To validate the performance of the proposed in-mouth antenna, the measurement was carried out using the minced pork and the saline solution for closed and opened mouth cases, respectively. The measured − 10 dB impedance bandwidth and peak gain values in the minced pork are from 2.28 to 2.53 GHz (10.39%) and − 18.17 dBi, respectively, and in the saline solution, are from 2.3 to 2.54 GHz (9.92%) and − 15.47 dBi, respectively. Further, the specific absorption rate (SAR) is estimated, and the data communication link is computed with and without a balun loss. This confirms that the proposed differential intraoral antenna can establish direct interfacing at the RF front end of the intraoral tongue drive system.

## Introduction

Wearable assistive devices are the most promising technologies in medical healthcare systems. It is uncertain and difficult to live with physical disabilities, which range from spinal cord injury (SCI) to tetraplegia damage^1–5^. These physical disabilities affect a vast number of people every year worldwide, with an average age of 29^2,6–8^. Tongue drive system (TDS) is a non-invasive and wearable assistive technology that allows users to operate and control nearby smart electronic devices (smartphone, laptop, powered wheelchair, etc.) using different tongue gestures^6,9^. The tongue is the most flexible organ that can potentially facilitate the rehabilitation of physically disabled people in the long run.

In the past, many TDS versions have been developed, external TDS (eTDS)^6^ and intraoral TDS (iTDS)^3,6,10–12^, taking into account their oral placements. An iTDS is the most preferred assistive technology adapted in two dental retainer forms: arch-shaped (a-iTDS)^10,11,13^ and palatal-shaped iTDS (p-iTDS)^3,10^. It comprises an array of sensor modules, control, and supply units integrated according to their proposed mouth placement to facilitate the transmission of the user’s intentions^11,12,14^. A system-on-a-chip (SoC), the brain of a dental retainer, is incorporated with other electronic components and properly attached to the upper teeth’s clasps so that they are well protected inside the mouth cavity (p-iTDS)^12,15^. In spite of the fact that iTDS-p is easier to build and use for earlier ATs^14,15^ and provides sufficient space for the electronics and battery, iTDS-a is preferable as it does not interfere with the intraoral space and offers the user more flexibility to generate a unique set of tongue instructions. Therefore, iTDS-a is considered for this research work, where we have designed an intraoral antenna that will facilitate the easy interfacing with other electronics integrated using commercial-off-the-shelf components (COTS) positioned on the mouth's buccal shelf area in front of lower teeth.

In arch-shaped iTDS, a magnetic tracer is temporarily fixed at the tip of the tongue. It generates magnetic fields for each tongue gesture which are sensed by a pair of magnetic sensors integrated along the left and right arm of the retainer. This retainer is positioned along the arch of lower teeth keeping in mind the arch-shaped iTDS configuration. The sensor’s information is then conditioned and processed using circuitry, which includes conditioning circuitry, an SoC microcontroller with a built-in 2.4-GHz RF transceiver (CC2510, Texas Instruments, Dallas TX), rechargeable batteries, and power management circuitry. This processed sensor signal is finally sent to the nearby smart electronic devices using an antenna integrated into iTDS and enables users to control them independently^6,11,13,16^.

**Figure 1:**
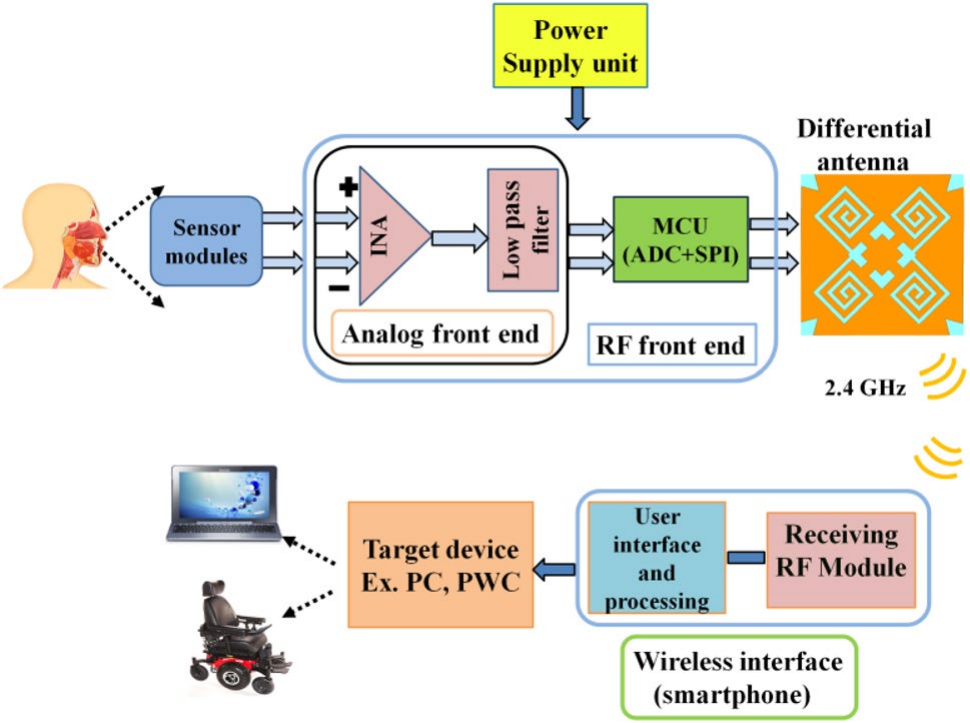
A high level high-level conceptual model of a differential wireless communication system for iTDS.

The tongue drive devices communicate tongue gestures using radio frequency (RF) front-end modules. These modules comprise compact antennas in order to facilitate easy interfacing and sustainable target access. A ten-wire flat cable that resembles a dental retainer connects the supply and control components^11^. Figure 1 shows the conceptual block-level diagram of the wireless differential tongue drive system. It can be seen that the physiological data from a set of left and right axial sensor modules are differentially fed at the analog front end. Consequently, this analog signal is adequately conditioned through two cascaded units, an instrumentation amplifier (INA) and a low pass filter (LPF). The microcontroller unit (MCU) further processes the conditioned analog signal before transmitting it wirelessly to the receiving device’s wireless interface. The wireless interface supports receiving information like a smartphone and USB module^11,17^. Furthermore, the tongue gesture information signal is wirelessly transmitted to external RF modules, where it is processed, displayed, and given to the target device, such as a PC or a PWC. It is important to note that the dual port controlling unit requires interfacing components such as Balun (impedance transformer) and matching circuits for single-fed antennas. However, a differential antenna that can be directly interfaced with the RF front-end modules due to its dual port configuration is a possible alternative^18,19^. So, the differential antennas must be considered to achieve both compactness and easy interfacing for the tongue drive devices^18,20^.

Wireless RF communication seems more appealing for intraoral devices owing to its ease of mobility and control. These devices commonly operate at the frequency of 2.45 GHz, as reported in Ref.^21^. In articles^3,11,21^, intraoral antennas have been proposed that work on a single frequency ISM band. In Ref.^3^, intraoral antennas were designed for palatal spacing based TDS. A T-shaped monopole and loop antennas were considered for encased devices with a size of 25 × 18 mm^2^. Although, it has been recommended that intraoral antennas integrated into arch-shaped TDS are preferred due to their privacy and maximum use of the oral cavity area. In Ref.^21^, a curved dipole antenna with a vertical length of 21.8 mm and radius of 0.3 mm was designed for arch-shaped TDS. It was operating only at the frequency of 2.4 GHz. An in-mouth antenna of size 60 × 13 mm^2^ was reported that was operating at 2450 MHz in the ISM band^11^. It was analyzed with system-level analysis considering both mouth scenarios for arch-shaped TDS. However, the planar size of antenna was large as compared to other intraoral antennas which will increase the overall volume of the tongue driven devices. Also, the SAR was not evaluated that provides quantitative measure of radiation absorbed by human tissues. It is important to estimate the SAR values for justifying user’s safety standards. In Ref.^22^, an intraoral antenna with a footprint area of 20 × 4.4 mm^2^ was proposed for the 915 MHz ISM band for arch-shaped TDS. Moreover, these intraoral antennas were not circularly polarized, which will increase the orientation-dependent losses. The data transfer mode of tongue drive devices with external RF modules is more effective with the use of circularly polarized (CP) antennas. It is possible due to limited line of sight (LOS) alignment issues between the transmitter and external receiving unit which further reduces the data transfer rate errors^23^. Furthermore, the previously reported TDS antennas have a critical need for extra matching circuits at the analog front end due to their unbalanced single-fed configuration, which will include additional losses and increase the complexity and size of the tongue drive devices^11,14^. For this direct interfacing, a differentially fed antenna is the best choice. A differentially fed antenna can facilitate direct interfacing and a good reduction in the overall size of the in-mouth tongue drive devices. Differential structured antennas can present strong immunity to environmental noise and direct interfacing with neighboring electronic circuits in TDS^24–26^.

The first differential antenna was reported for implantable medical device(IMD) suitable for intracranial pressure monitoring^18^. The planar size of the proposed antenna was 27 $\times$ 14 mm^2^. Spiral meandered slots were used to achieve resonance near the Medical Implant Communication Service (MICS) band (402–405 MHz). However, antenna biocompatibility was not analyzed in this study. Biocompatibility indicates the acceptance by adjacent human body tissues which are crucial for biomedical applications. In Ref.^27^, a differential antenna having the size of 34.5 × 5.8 mm^2^ was designed to fit in an ingestible capsule device. It provided resonance at a frequency of 0.915 GHz in the ISM band. In Ref.^28^, a circular differential antenna was considered with a planar size of $\pi \times {5}^{2}$ mm^2^ for wireless capsule endoscopy applications. It was operating at the higher frequency ISM band (2.4–2.525 GHz). However, the SAR parameter was not realized, which validates the user’s safety. Although, few research articles have presented the design of dual band differential antennas. In Ref.^29^, a novel dual band differential fed planar antenna was reported for near field biotelemetry applications. The square antenna was presented having the size of 23 × 22 mm^2^, which was achieved with the help of symmetrical meandered strip lines and shorting pins. But, the size of the antenna is not suitable for small implantable devices. In Ref.^25^, a differential implantable antenna was designed and integrated into an implantable device model for chest and head implantations. However, the use of a slotted ground plane and shorting pins could have been avoided to achieve a more robust antenna performance. It reveals that the reported differential antennas provide only linearly polarized radiation for in-body biomedical applications accounting for intracranial pressure monitoring^18^ and ingestible capsules^27,28^. Although, differential intraoral rectenna has been reported for the radiating near field power transfer system^26^. The work is detailed on differential intraoral antenna interfacing with a rectifier unit where the antenna comprises the full ground plane but at the cost of shorting pins. On the other hand, the single-fed antennas can also provide dual band operation with small dimensions but at the expense of additional interfacing losses^30,31^. In Ref.^31^, a single-fed intraoral antenna has been proposed for tongue driven system where the antenna design comprises a polyimide substrate sandwiched between meandered radiating patch and defected ground structure. Their single-fed geometry is not bound to symmetrically balanced structures and requires additional matching circuits. Please note that balanced structures are essential while designing the differential antennas to facilitate external noise cancellation and direct interfacing. Hence, in this paper, we have proposed a circularly polarized differential in-mouth antenna to work at a frequency of 2.4 GHz in the ISM band solely for wireless RF transmission of data attributed to iTDS application. The proposed in-mouth antenna comprises a full ground plane that minimizes harmful backward radiations. Additionally, the shorting pins have not been used to improve the tolerances that may appear in the fabrication process. Some basic information about biocompatible materials suitable for mouth environment exposure has been given. Thus, the proposed differential antenna can be a promising in-mouth antenna offering circularly polarized radiation without the need for defected ground structures and shorting pins.

Subject to the above discussion, this paper presents a compact circularly polarized differential in-mouth antenna for iTDS-based applications. The patch utilizes a central rotated square ring slot, and two pairs of spiral slots crossed diagonally that provide compactness. To achieve circular polarization, the proposed antenna uses pair of rotated spiral slots to introduce swirling of the electrical length and quadrature phase difference along the patch corners. The proposed antenna operates at 2.4 GHz ISM band and possesses a peak realized gain of – 17 dBi and – 14.15 dBi for closed and opened mouth environments. This band offers better compatibility due to the frequent use of commercialized RF transceivers in iTDS-based applications. To estimate the seamless data transfer range, the link budget is theoretically computed. The differential in-mouth antenna offers better performance by excluding the use of matching circuits and eliminating the additional matching circuit losses. Finally, the amount of acceptable power is calculated in terms of specific absorption rate (SAR) parameters based on IEEE standards. Table 1 shows the comparison of this proposed work with the previous studies. It is evident that the proposed antenna is delivering better performance in terms of low profile, CP radiation, peak gain, and estimated SAR.

**Table 1:** Performance comparison of the designed antenna vs. the previous studies.

Ref.	^21^	^3^	^29^	^11^	^32^		^28^	^31^		Proposed	
Publication (year)	2011	2015	2016	2018	2018		2021	2023		–	
Central freq. (MHz)	2450	2450	402$/$2400	2400	915, 2450		2400	433,2450		2400	
iTDS configuration	a-iTDS	p-iTDS	$\times$	a-TDS	$\times$		$\times$	a-iTDS		a-iTDS	
Antenna type	Wired dipole	Loop	Patch	Patch /PIFA	Patch		Patch	Patch		Patch	
Area (mm^2^)	Π × ${0.3}^{2}\times 21.8$	25 $\times$ 18	22 $\times$ 23	60 $\times$ 13	8 $\times$ 6		$\pi$×${5}^{2}$	10 $\times$ 10		13$. 1 \times$ 13.1	
Fractional BW (%)	–	3.3	7.4/6.6	18.8/29.2	9.84/8.57		10.2	65.5/27.7		10.39/9.92	
Gain (dBi)	$-$ 17 $.3 \left(C\right)/-$ 15.4 (O)	$-$ 21(C)	$-$ 36.7/− 27.1	$-14(C)/-10.6 (O)$	$-28.8/-22.8$		− 27.9	$-$ 37.5 $(C)/-$ 38.4 (O)	$-$ 35.2 $(C)/-$ 35.6 (O)	$-$ 17 $(C)/-$ 14.15 (O)	
Slotted ground	No	No	No	No	No		Yes	Yes		No	
Shorting pin	No	No	Yes	Yes	No		No	No		No	
Circular polarization	No	No	No	No	No		No	No		Yes	
Encapsulation material	Insulator (ε_r_ = 3.1 σ_e_ = 0 S/m)	Epoxy (ε_r_ = 3.5, σ_e_ = 0 S/m)	–	PDMS	Alumina (ε_r_ = 9.8)		Photosensitive resin (ε_r_ = 2.9, $\text{tan}\delta$ = 0.02 S/m)	Polyimide ε_r_ = 3.5, $\text{tan}\delta$ = 0.008 S/m)		PDMS	
SAR (W/kg) (1 − g/10-g avg.)	–	–	832/690 (1-g)	–	$971.56 /$807.34	$118.26/$102.04	–	$60.53 (\text{C})/$54.63 (O) (1 − g)	$92.19 (\text{C})/$80.95 (O) (1 − g)	$75.$69/20.37 (C)	$71.07/$17.19 (O)
Net input power (mW)	9.4/8.3	–	–	–	$971.56 /$807.34	$118.26/$102.04	–	$28.33 (\text{C})/$29.28 (O)	$19.64 (\text{C})/$19.67 (O)	$21.1/$98.2 (C)	$22.5/$116.3 (O)
Feeding topology	Single fed	Single fed	Differentially fed	Single fed	Single fed		Differentially fed	Single fed		Differentially fed	

*C* close mouth, *O* open mouth.

$\times$: not applicable; –: not given.

The authors have organized this manuscript into the following sections: “Methodology” section explains simulation mouth modeling in ANSYS High-Frequency Structure Simulator (HFSS), antenna design and geometry, and the evolution process of antenna design. It also contains some basic information on biocompatibility within the mouth environment. “Results and discussion” section discusses measurement setups (near field and far field) for differential reflection coefficient (|S_dd_|), axial ratio (AR), gain, and comparison of simulated and measured results. “Communication link” section provides an estimation of SAR and link budget. “Conclusion” section presents the conclusion of this work.

## Methodology

### Simulation setup for the proposed antenna

**Figure 2:**
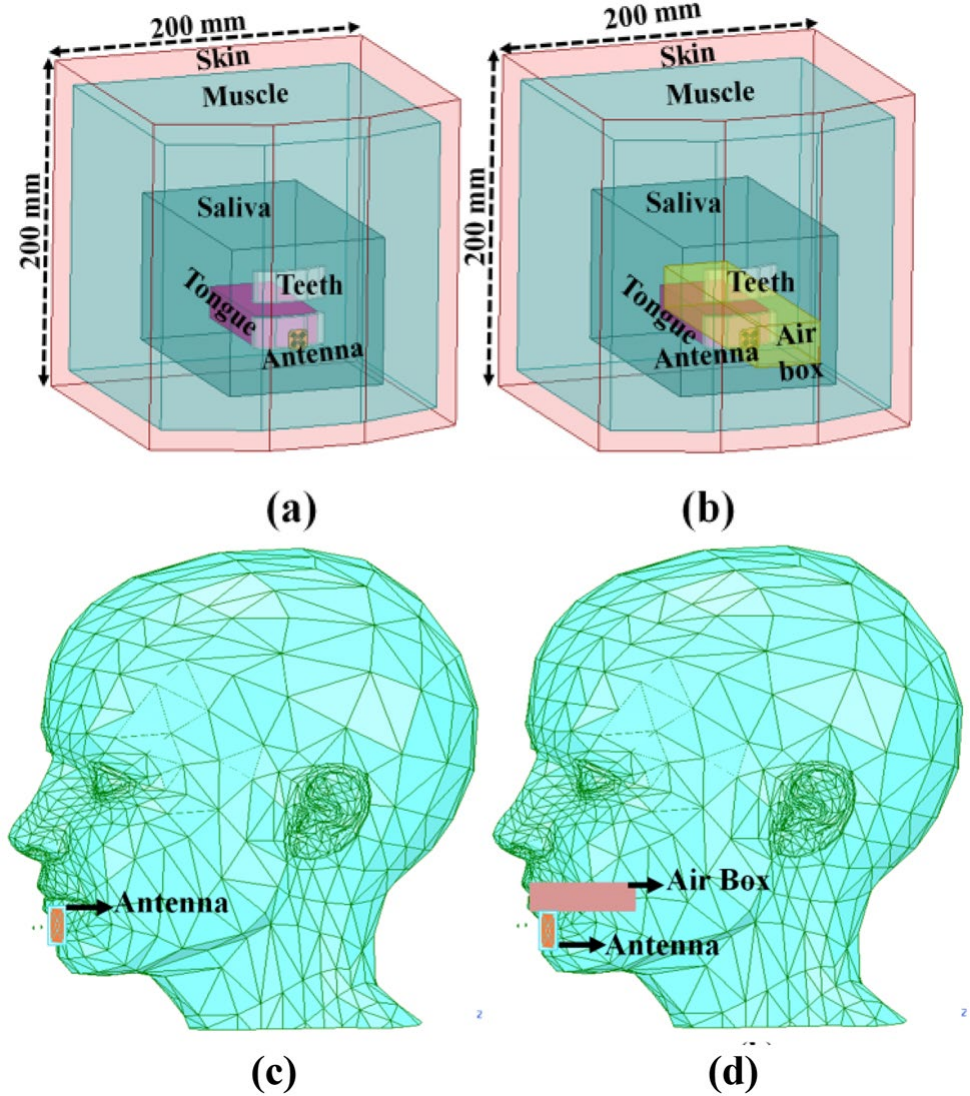
Oral anatomy placements of the proposed differential antenna in (**a**) multilayer oral cavity model (C), (**b**) multilayer mouth model (O), (**c**) averaged realistic closed human head model (C), (**d**) averaged realistic human head with an air box (O).

The Finite Element Method (FEM) based commercially available software HFSS.v.18 is selected to characterize the wireless communication channel. The proposed intraoral antenna design was initially analyzed in two scenarios (close and open mouth) using a multilayer heterogeneous mouth model with 200 mm × 200 mm dimensions in a prevalent 3D EM simulator (HFSS.v.18.0)^13^. The multilayer mouth model has been considered as a combination of different frequency-dependent tissues (skin, muscle, tongue, teeth, and saliva) for both scenarios, as depicted in Fig. 2a,b, and their electrical properties are presented in Table 2^11,33^. The antenna is proposed for arch-shaped iTDS, for which the antenna is assumed to be situated in front of the lower teeth in both scenarios. In the case of an open mouth, a box of dimensions 63 mm × 50 mm × 15 mm was embedded like an air retainer inside the multilayer mouth model, as depicted in Fig. 2b. Later, the performance of the designed antenna was also assessed using an averaged realistic human head model (HFSS.v.18) in both oral employments as reported in Fig. 2c,d: the averaged realistic closed human head model is represented in Fig. 2c, while Fig. 2d reports the model for an averaged realistic human head with an air box.

**Table 2:** Dielectric properties of mouth tissues and environment.

Tissues	2.4 GHz	
	*ε*_*r*_	*σ(S/m)*
Skin	38.06	1.44
Muscle	52.79	1.71
Saliva	74.1	1.07
Tongue	52.7	1.77
Teeth	11.4	0.385

### Circularly polarized differential intraoral antenna geometry and design

**Figure 3:**
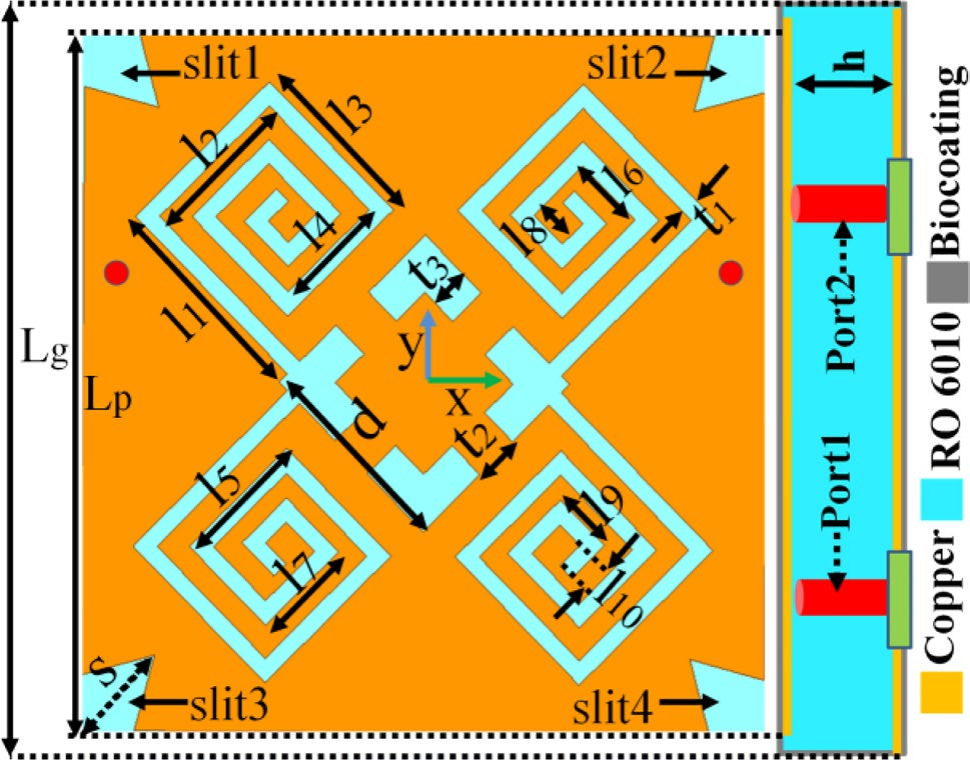
Geometry of the differential in-mouth antenna.

Pertaining to the non-static heterogeneous environment of the oral cavity, a circularly polarized differential antenna operating at 2.45 GHz is proposed in front of lower teeth for arch-shaped iTDS. The one-layered configuration (side view) with the geometrical design of the in-mouth antenna is presented below in Fig. 3. Table 3 lists the parameters and their values considered for the proposed antenna. The slotted radiating patch is balanced and symmetric about the x-axis providing easy interfacing at RF front end. The proposed antenna has been designed using Rogers RT/Duroid 6010 (ε_r_ = 10.2, tan δ = 0.0035), having a thickness of 0.635 mm to sustain the dynamic variations of the effective permittivity associated with different mouth tissues. On the front side, the patch utilizes a central rotated square ring slot and two pairs of spiral arms joined in diagonal form with the use of differential feeding. A full-ground plane was chosen on the backside to reduce the harmful backward radiations. It is highly recommended to encase the in-mouth antennas with a biocompatible material to improve their acceptability by surrounding tissues^34,35^. Here, the biocompatible polydimethylsiloxane (PDMS) encasing is chosen to protect the designed intraoral antenna from the body fluid (saliva) and improve the radiation efficiency of the in-mouth antenna. A 0.1 mm thick coating of PDMS has been wrapped all around the in-mouth antenna. The proposed encased in-mouth antenna has an overall volume of 145.49 mm^3^ (13.2 mm × 13.2 mm × 0.835 mm).

**Table 3:** Design parameters of an in-mouth antenna (step-4) (in mm).

Parameter	Value (mm)	Parameter	Value (mm)	Parameter	Value (mm)
*l*_1_	3.8	*l*_7_	1.8	*t*_1_	0.3
*l*_2_	2.7	*l*_8_	0.7	*t*_2_	0.7
*l*_3_	3.1	*l*_9_	1.1	*t*_3_	0.6
*l*_4_	2.0	*l*_10_	0.5	*s*	1.5
*l*_5_	2.4	*L*_*P*_	12	*h*	0.635
*l*_6_	1.3	*L*_*g*_	13	d	3.5

Some general considerations will be discussed to appreciate better why PDMS was used for the antenna encapsulation. Moreover, the conductivity of the antenna is proposed based on biocompatible alloys to reduce the eventual ion release or other corrosion effects as much as possible. During its lifetime, the proposed device, i.e., the intra-oral antenna, will be introduced in an actual oral environment, and for this reason, it is important to consider and spend some words about the safety of the materials since they will interact with the human tissue and corrosive liquids surroundings. Therefore, prior to its employment, some fundamental characteristics must be evaluated.

The first aspect is related to the presence in the oral cavity of multifaceted biological, chemical, and electrochemical agents. These elements are commonly additionally enriched by other organic compounds, like proteins, bacteria, etc., which give rise to a complex atmosphere that can also affect the lifetime of the device because of the deterioration of its surface. Furthermore, it can cause metabolite (lactate, glucose, glutamine, citrate, etc.) alterations, the risk for the patient, and end up with the possible failure of the device if not properly designed and realized. Additionally, over time pH modification (simply due to food consumption by eating and drinking) and temperature changes (during some diseases or consumption of food) undesirably affect the performance of the introduced medical tools, such as changes in the resonance frequency and/or bandwidth. The presence of saliva in the mouth is the essential carrier in such media, and it will extensively interact with the hosted devices^36,37^.

Even if, in this specific case, the presence of an antenna does not replace (neither totally nor partially) any part of the mouth, its presence in such an area can determine an improvement in the quality of people’s life. In this scenario, it is very important to consider and estimate how the antenna, considered a dental material, influences (or not) the correct evolution of the life cycle inside the mouth and, consequently, in the whole human body. Biocompatibility and the absence of cytotoxicity are some of the most important properties that materials used in the human body must complain about, and this aspect must be taken into consideration during the design. Evaluation of biocompatibility can be assessed both by in vitro and clinical studies, allowing us to obtain vital characteristics which are key elements during the design step of the devices and their efficacy^38–43^. Such study is over the topic of the present dissemination, but the authors have considered significant to underline their importance.

For differential feeding, a pair of the conventional terminals were fed with equal amplitude and 180° out-of-phase excitations to support the constructive interference of all differential mode (DM) signals and destructive interference of common mode (CM) signals. The differential feeding is realized using a pair of 0.5 mm probe diameter coaxial cables positioned at (± 5.2 mm, 2.1 mm). The reflection performance is evaluated in terms of corresponding reflection coefficients expressed as follows^44^:

1 $${S}_{dd11}={S}_{11}-{S}_{12},$$

2 $${S}_{cc11}={S}_{11}+{S}_{12}.$$

Also, the designed antenna demonstrates zero conversion of modes based on the balanced configuration of the in-mouth antenna, which results in the above simplified mathematical expressions.

### Evaluation process of differential intraoral antenna

**Figure 4:**
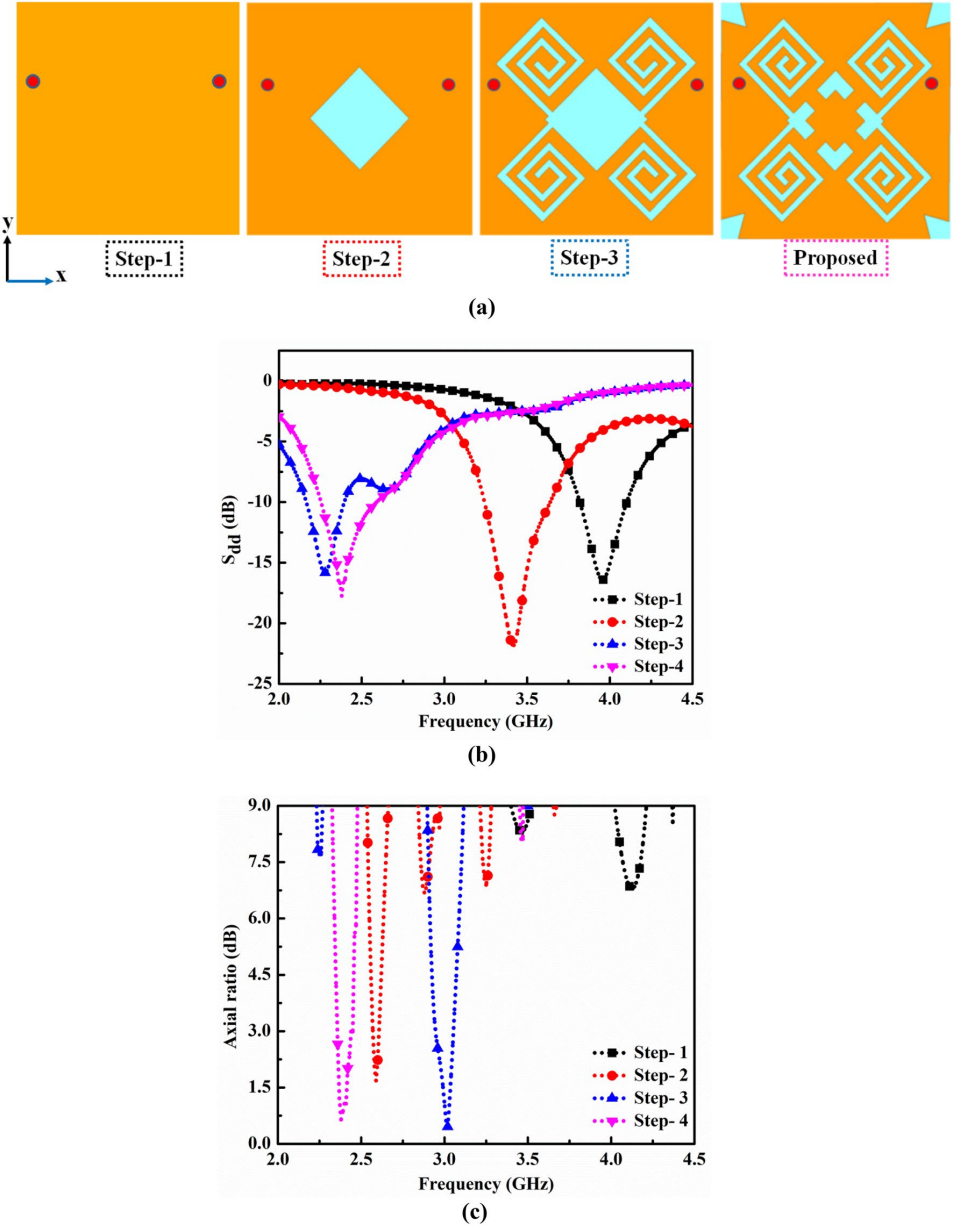
(**a**) Evolution stages of an in-mouth antenna design. (**b**) Differential mode reflection coefficients (S_dd11_), (**c**) axial ratios of each design step.

The four subsequent design stages, shown in Fig. 4a, represent the evolution of the antenna design. Figure 4b,c show the reflection coefficients and axial ratios corresponding to each design step. During the design evaluation process, some design goals have been considered. These goals are listed as (1) miniaturized design, (2) balanced geometry, (3) circular polarization, and (4) biocompatibility.

#### Design step-1 (the initial geometry)

Initially, a square patch of size 12 × 12 mm^2^ is considered with a fixed differential fed at (± 5.2 mm, 2.1 mm). The initial dimensions have been considered based on the Eq. (3):

$$f_{r} = \frac{c}{{L_{P2} \sqrt {\varepsilon_{eff} } }},$$

where, *c* is the speed of light in free space, *ε*_*eff*_ is the effective dielectric constant of the medium. Here it is equal to 6.65 according to the calculation with the microstrip line model. It has been discussed in detail in “Operation of the in-mouth antenna for radiation mechanism and circular polarization” section.

The corresponding resonance is found at a higher frequency of 3.79 GHz.

#### Design step-2 (rotated square slot)

A rotated square slot with side length, ‘d’, of 3.5 mm was loaded at the centre of the square patch, which introduced the capacitive effect and lowered the resonating frequency to 3.49 GHz with S_dd11_ of − 21.09 dB and a corresponding axial ratio less than 3 dB at the frequency of 2.59 GHz.

#### Design step-3 (diagonally loaded spiral slots)

Two pairs of spiral segments crossed in diagonal form were further loaded on the square patch to increase the current path's length. It shifted the frequency to 2.28 GHz, having an impedance matching of − 15.83 dB, and also increased capacitive reactance in the input impedance of the antenna design. Although, the pairs of spiral-shaped slots crossed in a diagonal manner kept an axial ratio of less than 3 dB. This could be possible because of the surface current twisting along the diagonally connected spiral-shaped slots and slightly shifting the frequency to 3.02 GHz.

#### Design step-4 (addition of a 45° rotated square stub and diagonal stubs in the radiating patch)

The rotated square slot was modified to the square ring slot by adding centrally a 45° rotated square stub and loaded with a 0.6 mm wide pair of diagonal stubs to form a pair of diagonally connected spirals. It helped to counter the capacitive effect of diagonally crossed spiral slots and sustain the circularly polarized radiation as well. The corner triangular slits were employed to tune the resonant frequency in the desired ISM band. The S_dd11_ (< 10 dB) and axial ratio (< 3 dB) were obtained as − 17.67 dB and 0.832 dB, respectively, at the resonating frequency of 2.38 GHz in the desired ISM band. It is to be noted that the proposed in-mouth antenna design in step-4 is symmetrical along the x-axis and thus enables balanced and easy interfacing with dual-port microwave devices.

### Estimation of in-mouth antenna performance in heterogeneous models

**Figure 5:**
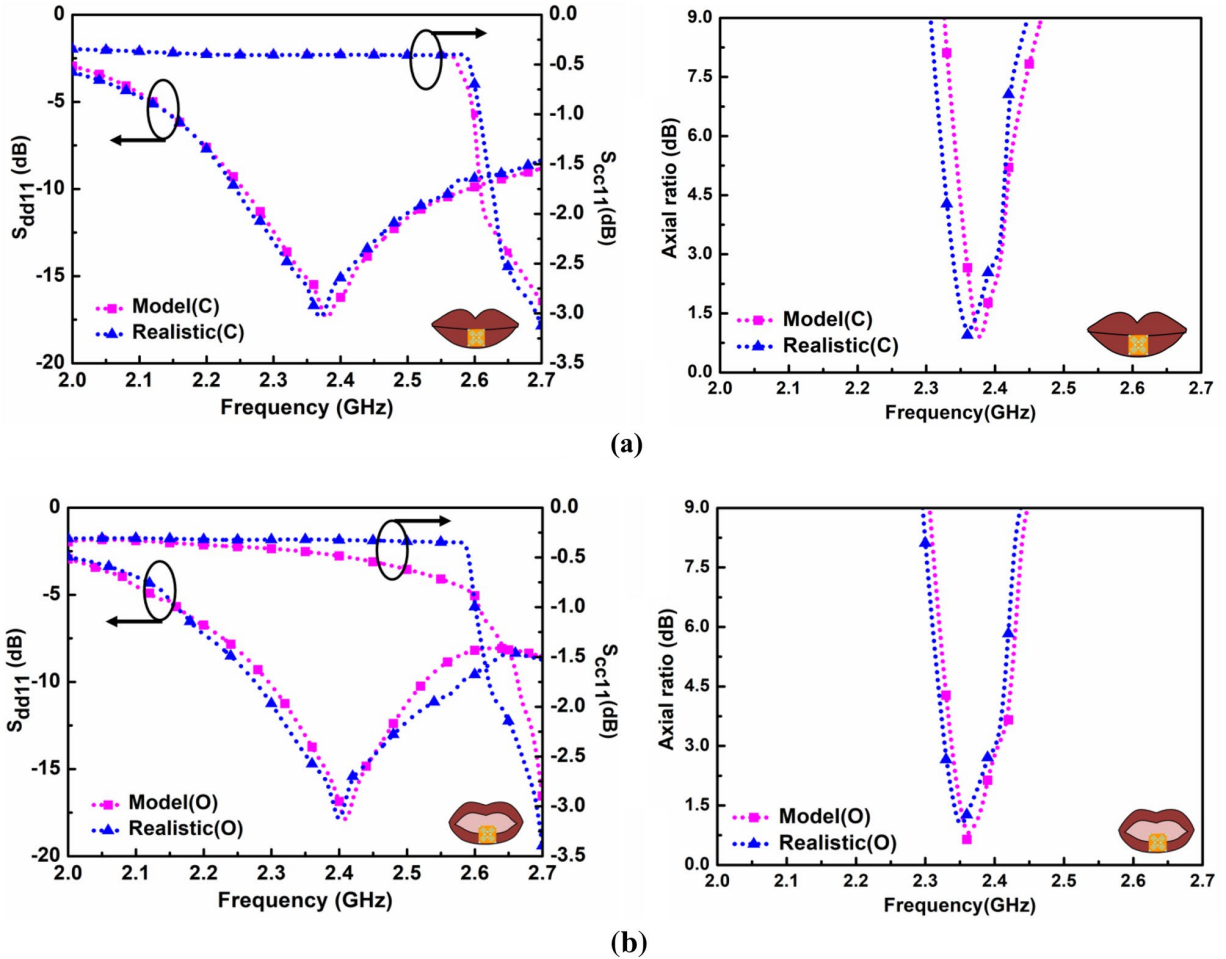
Simulated reflection coefficients (S_dd11_ and S_cc11_) and axial ratios of heterogeneous multilayer mouth models and realistic human head for (**a**) the closed and (**b**) opened mouth cases.

It can be seen from Fig. 5 that reflection coefficients are retrieved for differential mode and common mode signals exhibited as S_dd11_ and S_cc11_. These reflection coefficients are obtained for both the mouth cases in the multilayer simulation model and the realistic human head using Ansys HFSS.v.18. There is a slight shift in realistic models due to the asymmetric loading effect at higher frequencies. The values of the reflection coefficients associated with differential mode signals in close and open multilayer mouth models are − 17.670 dB and − 18.431 dB at 2.38 GHz and 2.4 GHz with impedance bandwidth (≤ − 10 dB) from 2.26 GHz to 2.56 GHz (12.45%) and 2.3 GHz to 2.57 GHz (11.08%). In contrast, in the realistic human head, these are − 17.710 dB and − 18.406 dB at (2.37 GHz and 2.39 GHz with impedance bandwidth (≤ − 10 dB) from 2.25 to 2.55 GHz and 2.28 to 2.57 GHz. In contrast, the values of the reflection coefficients associated with common-mode signals in close and open multilayer mouth models are − 0.403 dB and − 0.485 dB at 2.38 GHz and 2.4 GHz, whereas in the realistic human head, these are − 0.404 dB and − 0.322 dB at 2.37 GHz and 2.39 GHz. Similarly, the values of the axial ratios in close and open multilayer mouth models are 0.644 dB and 0.804 dB at 2.380 GHz and 2.365 GHz, whereas, in the realistic human head, these are 0.944 dB and 1.164 dB at 2.36 GHz and 2.35 GHz. It is observed that the simulation results of open-mouth cases are slightly better than in closed ones, which is attributed to the insertion of an air box. This specific area of the oral cavity is filled with air material in contrast to the closed mouth scenario, which compensates for the exposure of the in-mouth antenna to the dynamic surrounding fluid.

### Operation of the in-mouth antenna for radiation mechanism and circular polarization

In this section, the proposed in-mouth antenna’s radiation and circular polarization mechanism have been discussed at the resonating frequency of 2.4 GHz in the ISM band.

#### Radiation mechanism

**Figure 6:**
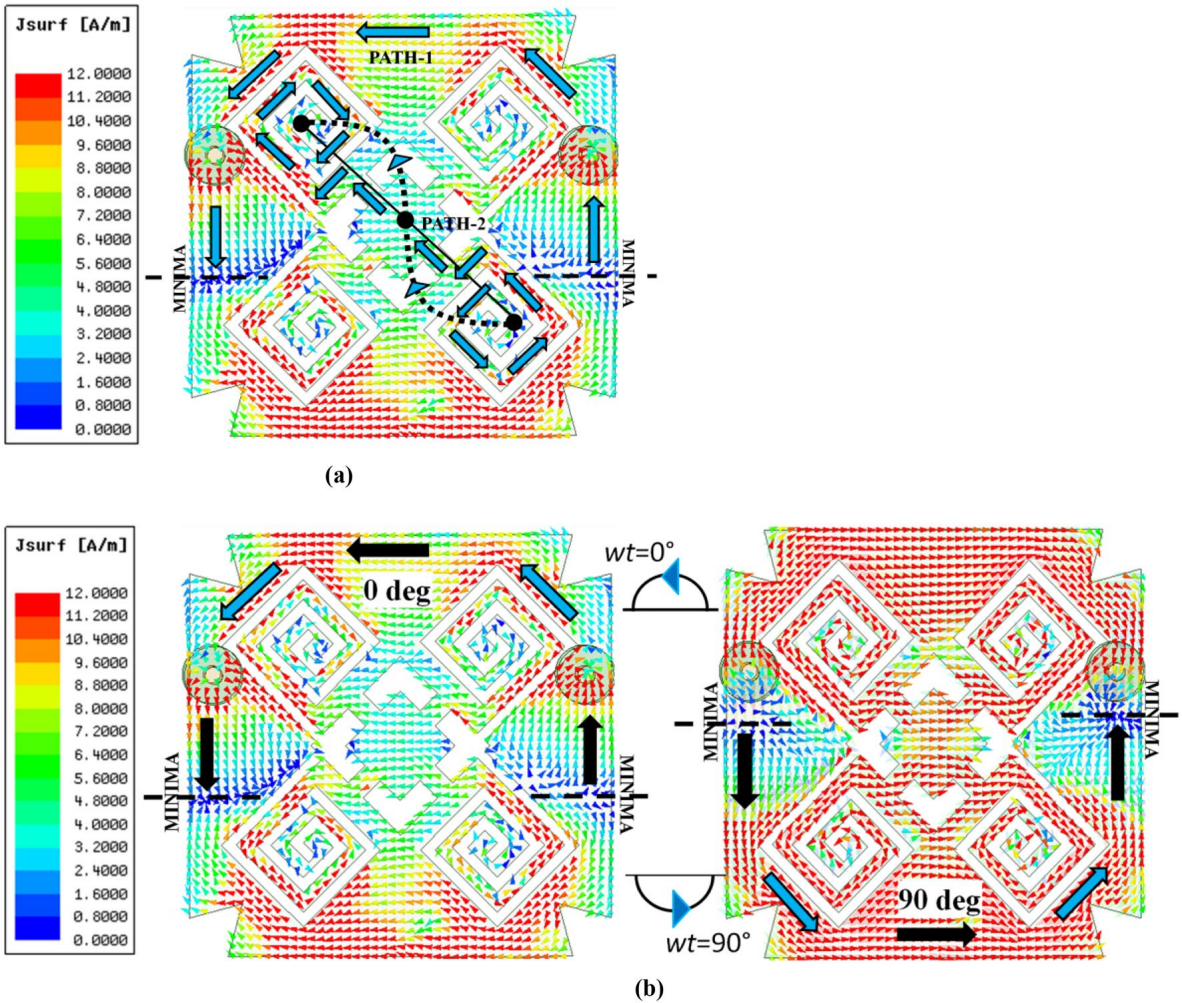
Surface current density distribution of proposed in-mouth antenna at 2.40 GHz for (**a**) Resonating path lengths, (**b**) time-phase, *ωt* = 0° and 90°.

It can be seen from Fig. 6a that the surface current transits over radiating patch from one half to the other half based on the potential gradient at the terminals of the proposed differential antenna along two paths. It is to be noted that the resonating length is calculated based on the surface current distributed around the rotating spiral arms, as shown in Fig. 6a. It is equivalent to the path length for Path-2, highlighted with black arrows, which is obtained as follows:

3 $${L}_{P2}=\left\{2\left[{l}_{1}+{l}_{2}+{l}_{3}+{l}_{4}+{l}_{5}+{l}_{6}+{l}_{7}+{l}_{8}+{l}_{9}+{l}_{10}+\left(\frac{d}{2}\right)\right]+\left(d\right)\right\}.$$

Based on boundary conditions, *L*_*P2*_ is equal to the guided wavelength. Hence the resonant frequency can be calculated as

4 $$f_{r} = \frac{c}{{L_{P2} \sqrt {\varepsilon_{eff} } }},$$

where, *c* is the speed of light in free space, *ε*_*eff*_ is the effective dielectric constant of the medium. Here it is equal to 6.65 according to the calculation with the microstrip line model. Likewise, the calculated L_*p2*_ is 46 mm. Therefore, the computed resonant frequency is 2.53 GHz. However, the numerically simulated resonance frequency is 2.4 GHz, which agrees with the computed resonant frequencies. It can also be seen that the full wavelength signal is nicely traversed from the right side to the left side along path-2 and attributed to the desired resonating frequency of 2.4 GHz.

#### Circular polarization

The proposed in-mouth antenna exhibits RHCP radiation when it is situated in front of lower teeth in a heterogeneous mouth model. Figure 4 depicts the simulated axial ratios for each evolution stage. A rotated square slot is loaded at the centre in design step-2, which provided the axial ratio (< 3 dB) around the frequency of 2.62 GHz. This rotated square slot induces the quadrature phase difference in the surface current along its edges. To keep the axial ratio intact, pairs of spiral slots and stubs (a rotated square stub of side ‘($$d-{t}_{3})$$’ mm and a pair of ‘$${t}_{2}$$’ mm wide stub) are loaded diagonally with respect to the centre rotated square slot in the design step-3 and 4, respectively. The diagonally loaded spiral slots and stubs incorporated phase quadrature at 2.4 GHz for two adjacent time phases, ωt = 0°, 90°. To further visualize the CP performance, the surface current distribution of ‘path-1’ is studied for two adjacent time phases, as shown in Fig. 6b.

At ωt = 0°, it is possible to observe the rotation of a half-wavelength current moving from the patch’s right to left side depicted using blue and black arrows. As the time phase advances by a quarter of wavelength (ωt = 90°), the two minima (dashed line) align so that a second half-wavelength current moves in a circle from the patch’s left side to the right. It can be observed that the blue current vector is equivalent to the sum of orthogonal black vectors in each half of the proposed antenna at two adjacent time phases. These alternate rotations of half-wavelength currents contribute to an anticlockwise revolving electric current which shows that the proposed in-mouth antenna could present RHCP radiation at 2.40 GHz.

**Figure 7:**
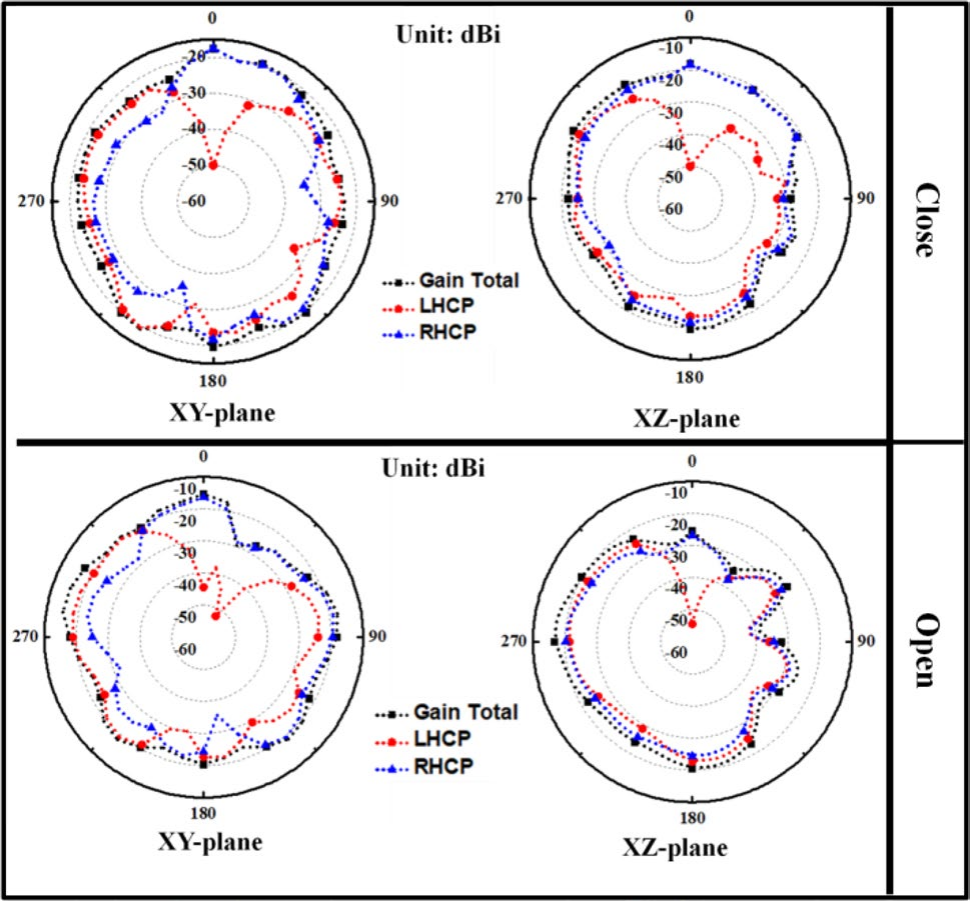
Simulated far-field radiation patterns at 2.4 GHz in the close and open-mouth scenarios (Multilayer mouth model).

Figure 7 shows the simulated radiation patterns (for the multilayer mouth model) of the proposed differential antenna in azimuth (XY-plane) and elevation (XZ-plane) planes at 2.4 GHz for corresponding closed- and open-mouth scenarios. It can be seen that this intraoral antenna can effectively radiate RHCP in the boresight direction. The level differences between the simulated RHCP (*co-polarization*) and LHCP (*cross-polarization*) are more than 15 dB for both mouth cases. It also validates that the proposed antenna is well capable of producing co-polarized RHCP waves. The peak gains observed in the multilayer human mouth model were − 17 dB and − 14.15 dB for the closed and open-mouth cases, respectively. It can be seen from Fig. 7 that the proposed antenna is propagating effectively outside the mouth, which is important for data communication of iTDS-based applications.

### Equivalent circuit model

**Figure 8:**
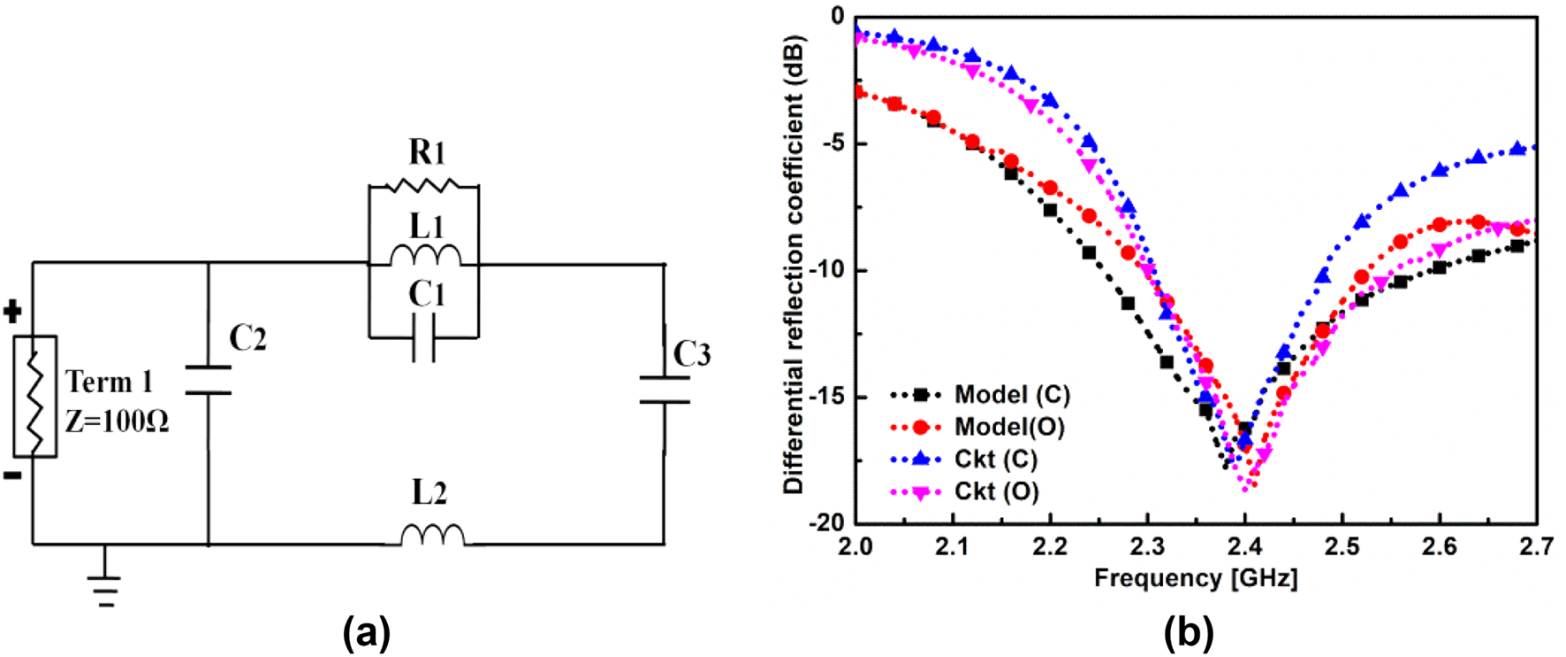
(**a**) Equivalent circuit model of the proposed differential in-mouth antenna. (**b**) Simulated odd mode reflection coefficients (Using HFSS and ADS).

The proposed in-mouth antenna has been characterized as a 100 Ω terminal to facilitate the analysis of the differential topology of the proposed antenna. The proposed antenna’s lumped element equivalent circuit model, in step 4, has been designed with the help of an advanced design system (ADS), shown in Fig. 8a. The two pairs of diagonal spiral slots loaded near the edges of the central rotated square slot on the radiating patch provided desired frequency resonance in the closed and open mouth scenarios. Therefore, a parallel connected RLC (tank) circuit is modeled for the corresponding f resonant frequency^45^. Here, a parallel connection of R1, L1, and C1 employs the frequency resonance, where R1 denotes the radiation resistance of the RLC tank circuit. Also, C2 and C3 constitute the capacitive coupling effect along radiating edges^46^, whereas L2 amounts to the ground inductive reactance. The corresponding values of lumped components have been obtained using the standard equations of RLC tank circuits and later optimized for desired resonance in ADS as given in Table 4. Figure 8b depicts the comparison of simulated differential reflection coefficients of the designed in-mouth antenna derived using 3D simulators, HFSS and ADS. It is observed that the differential reflection coefficient of the proposed antenna and circuit model closely match each other in the ISM frequency band.

**Table 4:** Component values in closed and opened mouth cases.

Frequency (GHz)	R1 (ohm)	L1 (nH)	C1 (pF)	L2 (nH)	C2 (pF)	C3 (pF)
2.38 GHz **(C)**	142	1.16	3.36	15.0	0.45	0.26
2.40 GHz **(O)**	118	1.17	3.35	15.7	0.38	0.26

## Results and discussion

### Near-field measurement

**Figure 9:**
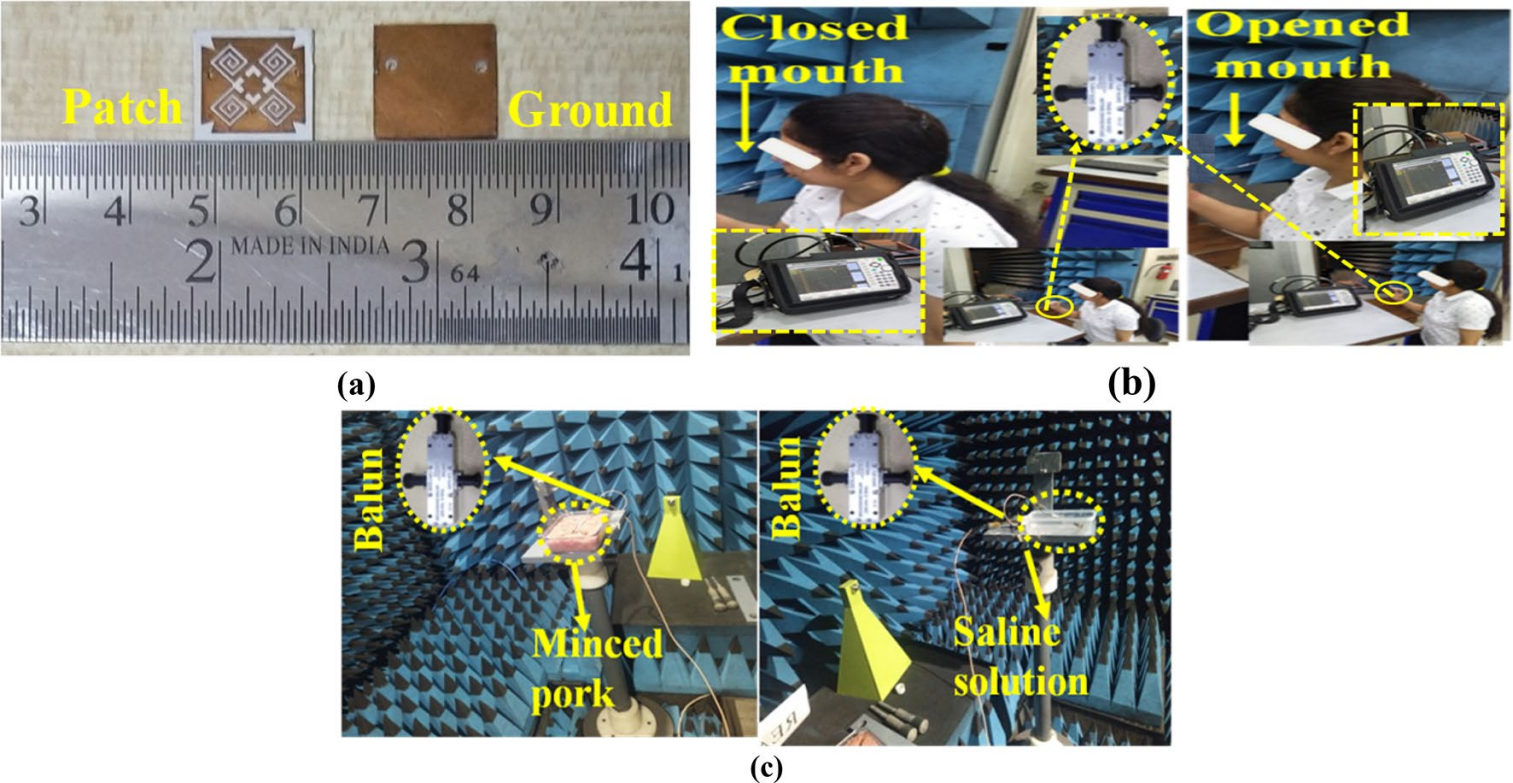
(**a**) Fabricated in-mouth differential antenna (top and back view), (**b**) near field measurement setup, (**c**) far field measurement setup with a minced pork and saline solution for closed and opened mouth approximation, respectively.

The proposed in-mouth antenna was manufactured on the RO 6010 substrate and encased with a 0.1 mm thick polyamide layer for experimental validation, as shown in Fig. 9a. The reflection performance was evaluated using Anritsu MS2038C, a two-port vector network analyzer, in the human subject’s mouth, for both opened and closed mouth scenarios, as presented in Fig. 9b. The front two cables are first calibrated separately, utilizing the SOLT (short, open loads, and through ports) calibration kit. The desired differential reflection coefficient (S_dd_) was measured using a Balun (part: BAL-0006 from Marki Microwave), which provides impedance transformation of 100 Ω (input terminals) to 50 Ω (output terminal) differential impedance transformation as depicted in Fig. 9. The Balun served as an impedance transformer for converting 100 Ω (input terminal) to 50 Ω (output terminal) for both mouth scenarios^47,48^. Additionally, the open-ended coaxial probe approach was utilized to assess the dielectric characteristics of homogeneous phantoms and tissues^49^.

**Figure 10:**
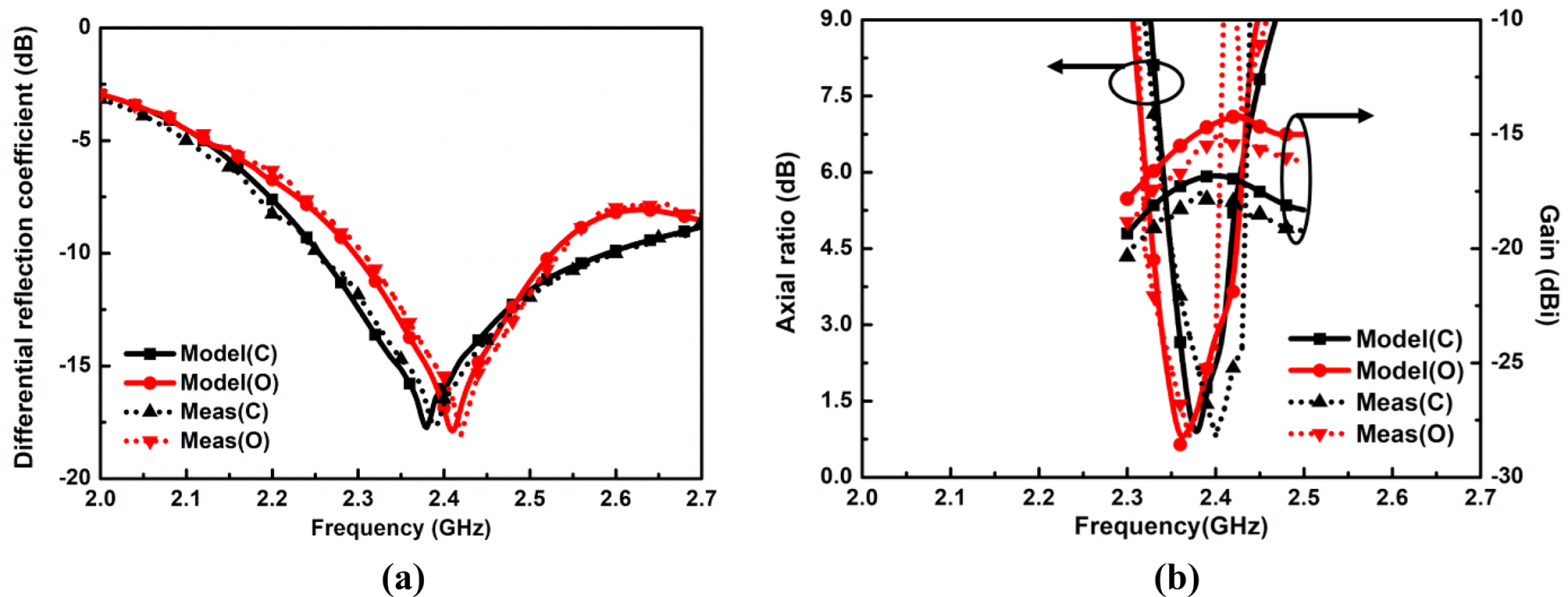
Simulated and measured (**a**) Differential reflection coefficients. (**b**) Axial ratios and gain values of the proposed in-mouth antenna.

Figure 10 depicts the comparison of simulated and measured differential reflection parameters, axial ratios, and peak gain of the proposed in-mouth differential antenna. The measured impedance bandwidth in muscle phantom and solution-filled artificial head models are from 2.28 to 2.53 GHz (10.39%) and 2.3 to 2.54 GHz (9.92%) at 2.40 GHz.

### Far-field measurement

**Figure 11:**
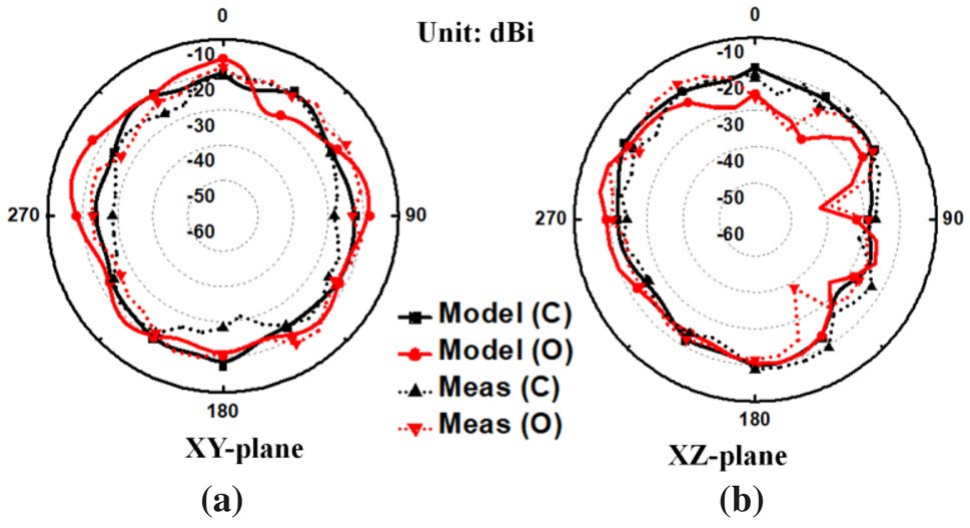
Simulated and measured far-field radiation patterns of the proposed in-mouth antenna in (**a**) XY /azimuthal, (**b**) XZ/elevation planes.

In the far-field measurement scenario, the proposed in-mouth antenna was positioned at the rotating stand as a receiver which was fixed at a distance of 310 cm from the transmitter. In this measurement setup, the horn antenna (DRH20) was used as a transmitter with an input power of 0 dBm. The measurement of far-field gain was driven again using a balun interface, which performs the conversion of double-ended signals into single-ended signals, which are fed to the end spectrum analyzer, as depicted in Fig. 9b. Measurements were carried out inside an anechoic chamber using minced pork and saline solution to replicate the conditions in both mouth cases. For close-mouth employment, the antenna was inserted into the minced pork in a tiny cavity that was 3 mm deep, as depicted in Fig. 9c. In the case of open-mouth employment, the in-mouth antenna was situated in a saline solution filled rectangular container, as shown in Fig. 9c. Later, the peak values of the measured gain are computed using the standard Friis equation. Notably, the proposed in-mouth antenna could feed the differential dual port microwave circuits directly, eliminating the requirement for a balun for the final real TDS application. The values of peak gains are − 18.17 dBi, and − 15.47 dBi for closed and opened mouth cases, respectively. The calculated peak gains are close to the simulated results, and the measured axial ratios slightly drift to upward frequencies in both mouth cases. In both mouth cases, the measured results of the axial ratio somewhat shift to a higher frequency, and the estimated peak gains are marginally less than the simulated peak gains. It can be seen from Fig. 11 that the measured and simulated far-field gain radiation patterns are in close agreement in both planes. The small discrepancies are acceptable, which are present due to the fabrication errors and approximate mouth scenarios. But, the measured results are well close to the simulated results.

## Communication link

### SAR analysis

**Figure 12:**
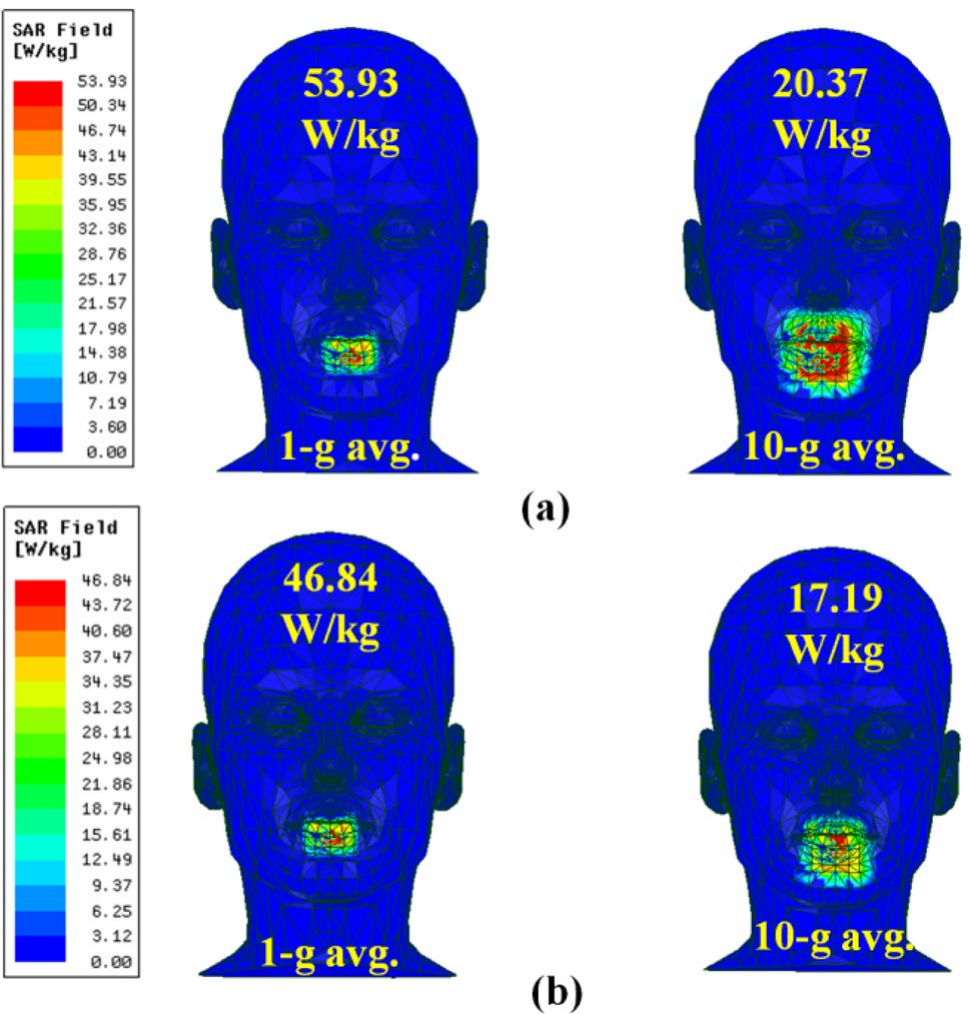
Simulated average SAR distributions over 1-g and 10-g of tissue at 2.4 GHz for (**a**) Close mouth, (**b**) open mouth.

For user safety with respect to SAR values, two commonly used standards are IEEE C95.1-1999, and IEEE C95.1-2005, which provide average SAR limits over 1 g and 10 g of tissue to less than 1.6 W/kg and 2W/kg, respectively. Figure 12 depicts simulated average SAR distributions over 1-g and 10-g of tissue at 2.4 GHz for close and open-mouth cases (female human head). Table 5 presents the proposed intraoral antenna's simulated maximum SAR and maximum input power for open and closed-mouth scenarios at 2.4 GHz. The maximum net input power over 1-g and 10-g of tissue was observed to be 34.15 mW and 116.34 mW. The input power restriction for in-body devices is 25 µW, so, based on this estimation, the maximum SAR of the proposed antenna would not be a serious concern.

**Table 5:** Maximum SAR and allowable input power values at 2.40 GHz.

Mouth scenarios	Avg SAR (W/kg)		Max net input power (mW)	
	1-g	10-g	1-g	10-g
Close	53.93	20.37	29.67	98.18
Open	46.84	17.19	34.15	116.34

### Link budget analysis

The wireless communication range of the proposed differential in-mouth antenna is assessed with an exterior base station by link budget analysis. For the data telemetry link between the proposed in-mouth antenna (*T*_*x*_) and external antenna (*R*_*x*_), the link budget is theoretically computed at two different data transfer rates (*B*_*r*_) for opened and closed human mouth scenarios using the standard equations in Ref.^50^. The transmitter power level (*P*_*t*_) is fixed at − 16dBm (25 μW), which is the maximum permissible emitted power (EIRP) for wireless body area networks (Wi-Fi Bluetooth, IoTs, etc.), and receiver half-wave dipole antenna gain (*G*_*r*_) is considered as 2.15dBi.

For TDS antennas^11,14^, the requirement of a balun interface is unavoidable because of the dual port microcontroller unit (CC2510) at both the transmitting and receiving ends in the final TDS application. Concerning the balanced interfacing at the analog front end, the link margin is estimated in terms of the integration of Balun through two scenarios, with/without Balun. Here, the balun losses are assumed from Ref.^11^. Table 6 shows all the parameters considered during the estimation of the link budget at 2.4 GHz.

**Table 6:** Link budget parameters.

Transmitter		
f_r_ (GHz)	Resonating frequency	2.40
P_t_ (dBW)	Transmitted power	− 46.0
G_t_ (dBi)	In-mouth antenna gain	− 17.00 (C)/− 14.15 (O)
EIRP	Effective isotropic radiated power	− 33.00/− 30.15
ML_TX_(dB)	Mismatch loss	0.07/0.062
Propagation		
L_f_ (dB)	Free space loss	Distance dependent
Receiver		
G_r_ (dBi)	External antenna gain	2.15
T_0_ (Kelvin)	Temperature	273
K	Boltzmann constant	1.38E-23
N_0_ (dB/Hz)	Noise power density	− 203.9
ν	Linear axial ratio	1.41(C)/1.43(O)
Lp (dB)	Polarization loss (α = 0° = 180°)	0.81/0.74
	(α = 90° = 270°)	7.72/8.04
Signal quality		
B_r_ (Kb/S)	Bit rate	250
E_b_/N_0_ (dB)	PSK	9.6
G_c_ (dB)	Coding gain	0
G_d_ (dB)	Fixing deterioration	2.5

**Figure 13:**
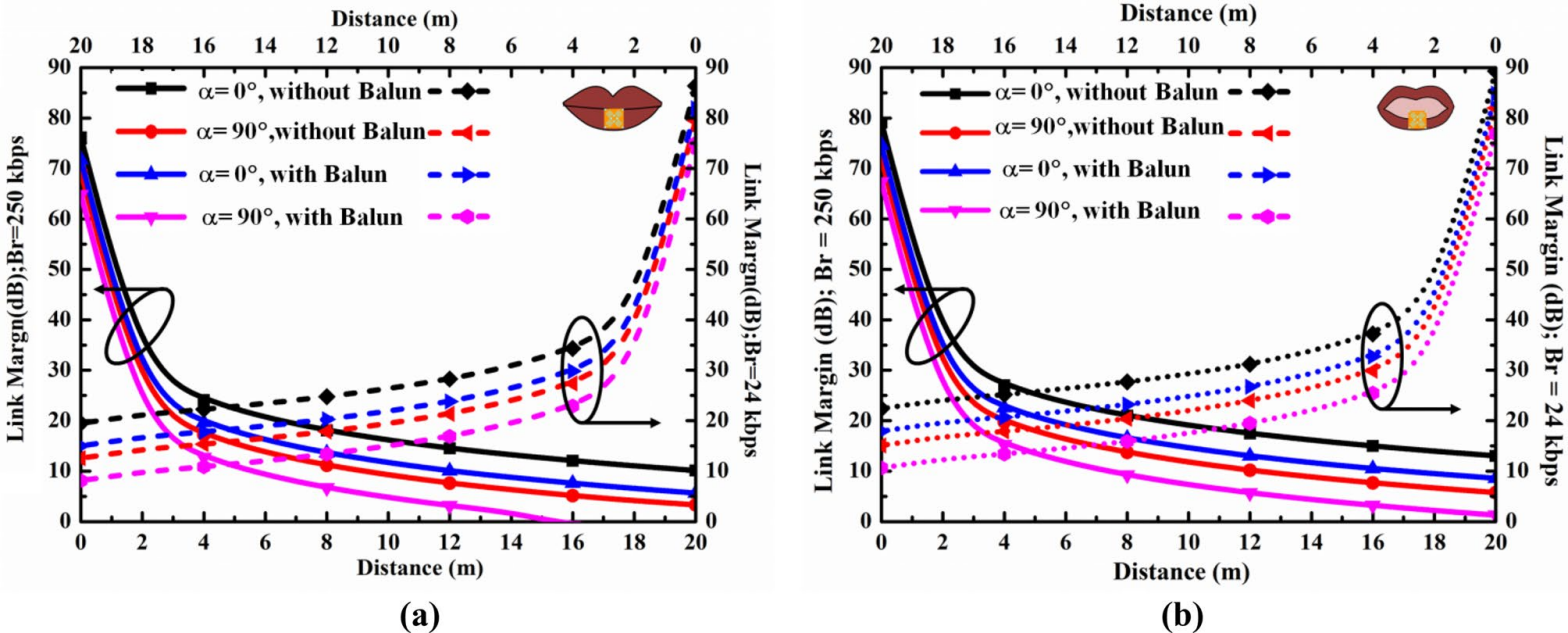
Calculated link margin of the proposed intraoral antenna at different data rates for (**a**) closed mouth, (**b**) opened mouth.

As it can be noticed, |Sdd| values of the in-mouth differential antenna are − 17.670 dB and − 18.431 dB for closed and opened mouth cases at 2.40 GHz, respectively (see Fig. 5). The corresponding impedance mismatch losses are obtained using a mathematical expression from Ref.^50^ and listed in Table 6. Also, the polarization mismatch losses have also been calculated for both the mouth scenarios considering various angles of the receiving antenna (α) by taking the equation reported in Ref.^51^. It is recommended that the minimum link margin of 10 dB is enough to drive the data transfer from the user’s device to the exterior target^50^. For a data transfer rate of 250 kbps considering with/without balun cases, the in-mouth differential antenna could effectively transfer sensor data up to 20 m/14 m, at α = 0° = 180°, and 10 m/6 m, at α = 90° = 270° when exposed to closed mouth environment (Fig. 13b). On other hands, for the data transfer rate of 24 Kbps, the in-mouth differential antenna could efficiently communicate the biological data beyond the distance of 20 m at α = 0° = 180° whereas it gets reduced to communication range up to 16 m (with Balun) at α = 90° = 270°. In the case of opened mouth environment (Fig. 13b), it could transfer data rate of 250 Kbps beyond and up to 20 m, at α = 0° = 180° and 14 m/8 m (with/without Balun) at α = 90° = 270°. However, for a data rate of 24 kbps, it could transfer the data beyond 20 m at all angles considering both balun cases. It can be seen from Fig. 13 that an additional loss of 4.4 dB has occurred in the case of Balun (at α = 0° and 90°) at different data transfer rates in both the mouth cases, which can significantly degrade the communication capacity of the proposed antenna. It is to be noted that the differential in-mouth antenna could eliminate the additional loss of the matching interfaces (Balun) due to its direct connection with the dual port MCU of iTDS. Also, the minimum distance between the subject and PWC/PC is supposed to be 70 cm/51 cm. It can be inferred that the proposed differential in-mouth antenna can transfer the biological data over a reasonable distance by overcoming the need for Balun, as illustrated in Fig. 13a,b. Figure 13 shows that the proposed antenna can communicate effectively up to 20 m at a data rate of 24 kbps with an input power of 25 μW and 20 dB link margin for both mouth scenarios (open and closed). For a higher data rate (250 kbps), the communication range can be a maximum of up to 8 m following a higher 20 dB link margin. Although, the link margin of 10 dB is considered enough for reliable communication from the internal device to the exterior unit^10,50^. Hence, the proposed differential in-mouth antenna would prove its stronghold in serving navigational iTDS-based data-telemetry applications at different data rates.

## Conclusion

A circularly polarized differential in-mouth antenna was proposed, designed, and validated at 2.40 GHz in the ISM band for TDS-based applications. The 2.40 GHz ISM band offers better compatibility due to the frequent use of commercialized RF transceivers in iTDS-based applications with respect to other bands. The fabricated prototype of the proposed differential in-mouth antenna occupies a small volume of 145.49 mm^3^ (13.2 mm $\times$ 13.2 $\text{mm} \times$ 0.835 mm). High permittivity substrate Rogers RT/ Duroid 6010 (ε_r_ = 10.2, tan δ = 0.0035), having a thickness of 0.635 mm, is opted to sustain the dynamic variations of the effective permittivity associated with different mouth tissues. Biocompatible encasing of a polydimethylsiloxane (PDMS) is used to protect the designed intraoral antenna from the body fluid (saliva) and improve the lifetime of the hosted tongue drive device. A central rotated square ring slot and two pairs of spiral slots crossed diagonally have been introduced on the radiating patch to keep the proposed antenna size compact. To excite circularly polarized radiation, the proposed antenna has opted a pair of rotated spiral slots in order to introduce swirling of the electrical length and the direction of the flowing current. The antenna was analyzed in two simulation environments approximated for open and closed-mouth scenarios using human mouth models and a realistic human head in a 3D EM simulator (Ansys HFSS.v.18). The measurement of the fabricated prototype of the antenna was carried out in the muscle phantom, and solution filled artificial head model to approximate open and closed mouth cases. For open and closed-mouth placements, the measured realized peak gain values were − 18.17 dBi and − 15.47 dBi. Further, the capability to transfer data rates (24 and 250 kbps) with input power (25 uW) well conforming to SAR limits validates the proposed antenna for reliable communication in iTDS. Also, it can facilitate easy interfacing with dual port MCU within RF transceivers and eliminate the need for extra matching circuits, which is theoretically assessed in link budget analysis. It can further channelize communication over wireless body area network IoTs through smart wireless interface technologies. In future investigations, the flexibility impact of different TDS biocompatible materials will be studied. Additionally, different antenna designs using Rogers will be realized.
